# Enhanced ZAG production by subcutaneous adipose tissue is linked to weight loss in gastrointestinal cancer patients

**DOI:** 10.1038/sj.bjc.6606083

**Published:** 2011-01-18

**Authors:** T Mracek, N A Stephens, D Gao, Y Bao, J A Ross, M Rydén, P Arner, P Trayhurn, K C H Fearon, C Bing

**Affiliations:** 1 Obesity Biology Research Unit, School of Clinical Sciences, University of Liverpool, Liverpool L69 3GA, UK; 2 Department of Clinical and Surgical Sciences (Surgery), University of Edinburgh, Edinburgh, UK; 3 Department of Medicine, Karolinska Institutet at Karolinska University Hospital, Stockholm, Sweden

**Keywords:** zinc-*α*2-glycoprotein, gastrointestinal cancer, cachexia, adipose tissue, lipolysis

## Abstract

**Background:**

Profound loss of adipose tissue is a hallmark of cancer cachexia. Zinc-*α*2-glycoprotein (ZAG), a recently identified adipokine, is suggested as a candidate in lipid catabolism.

**Methods:**

In the first study, eight weight-stable and 17 cachectic cancer patients (weight loss ⩾5% in previous 6 months) were recruited. Zinc-*α*2-glycoprotein mRNA and protein expression were assessed in subcutaneous adipose tissue (SAT), subcutaneous adipose tissue morphology was examined and serum ZAG concentrations were quantified. In the second cohort, ZAG release by SAT was determined in 18 weight-stable and 15 cachectic cancer patients. The effect of ZAG on lipolysis was evaluated *in vitro*.

**Results:**

Subcutaneous adipose tissue remodelling in cancer cachexia was evident through shrunken adipocytes with increased fibrosis. In cachectic cancer patients, ZAG mRNA was upregulated (2.7-fold, *P*=0.028) while leptin mRNA decreased (2.2-fold, *P*=0.018); serum ZAG levels were found to be unaffected. Zinc-*α*2-glycoprotein mRNA correlated positively with weight loss (*r*=0.51, *P*=0.01) and serum glycerol levels (*r*=0.57, *P*=0.003). Zinc-*α*2-glycoprotein release by SAT was also elevated in cachectic patients (1.5-fold, *P*=0.024) and correlated with weight loss (*r*=0.50, *P*=0.003). Recombinant ZAG stimulated lipolysis in human adipocytes.

**Conclusions:**

Zinc-*α*2-glycoprotein expression and secretion by adipose tissue is enhanced in cachectic cancer patients. Given its lipid-mobilising effect, ZAG may contribute to adipose atrophy associated with cancer cachexia in human beings.

Cachexia, a catabolic syndrome manifested by profound loss of adipose tissue and skeletal muscle, affects most cancer patients (Fearon *et al*, 2006). Progressive cachexia has a detrimental effect on antitumour treatment and is associated with reduced survival (Dewys *et al*, 1980). Muscle wasting has been the focus of intensive research, with the demonstration of reduced protein synthesis and increased proteolysis in rodent models of cachexia and in cachectic cancer patients (Tisdale, 2005; Argiles *et al*, 2006). Fat loss occurs more rapidly than the reduction of lean mass in cancer cachexia (Fouladiun *et al*, 2005), with the most rapid loss of adipose tissue within 3 months of death (Lieffers *et al*, 2009). Although extensive depletion of body fat has been considered as a hallmark of cancer cachexia, the underlying molecular basis is poorly understood. A better knowledge of the mechanisms involved in adipose atrophy is crucial for the development of effective treatments for the syndrome.

Several factors derived from tumours and/or host tissues are implicated in fat depletion during progressive cachexia (Tisdale, 2005). These factors include inflammatory cytokines such as tumour necrosis factor *α* (TNF*α*) and lipolytic factors such as zinc-*α*2-glycoprotein (ZAG). Zinc-*α*2-glycoprotein, a secreted soluble protein, was initially isolated from human plasma (Burgi and Schmid, 1961), and subsequently found in various tissues (Tada *et al*, 1991). Zinc-*α*2-glycoprotein is overexpressed by several types of malignant tumour, such as breast, prostate and lung cancers (Diez-Itza *et al*, 1993; Hale *et al*, 2001; Albertus *et al*, 2008), and it has been proposed as a cancer biomarker. The physiological functions of ZAG remain to be established, but the protein has been implicated in hindering cell proliferation (He *et al*, 2001; Schmitt *et al*, 2008) and modulating melanin production (Hale, 2002).

The most studied property of ZAG is its involvement in lipid metabolism (Bing and Trayhurn, 2009). Zinc-*α*2-glycoprotein has been shown to be identical to a lipid-mobilising factor (LMF) purified from the urine of patients with cancer cachexia (Todorov *et al*, 1998). Treatment with purified ZAG can induce selective reduction in body fat in both genetically obese (*ob/ob*) mice and outbred NMRI mice (Hirai *et al*, 1998; Bing *et al*, 2002). *In vitro*, ZAG stimulates lipolysis in isolated murine and human adipocytes, and this has been postulated to be mediated by the *β*_3_-adrenoceptor in rodents (Hirai *et al*, 1998). There is also evidence that ZAG promotes lipid utilisation through the upregulation of uncoupling protein-1 in brown fat (Bing *et al*, 2002) and increasing overall fatty acid oxidation (Russell and Tisdale, 2010).

Adipose tissue plays important roles in energy homeostasis and metabolism through secreted adipokines (Trayhurn and Bing, 2006). The fat-lowering effect of ZAG and the secretory function of adipose tissue led to the hypothesis that ZAG could be produced by adipose tissue, thereby influencing adipocyte metabolism (Bing *et al*, 2004). Indeed, our previous work has shown that the *ZAG* gene and protein is expressed in the major fat depots of mice and human beings (Bing *et al*, 2004). Subsequently, ZAG was shown to be secreted by differentiated human adipocytes (Bao *et al*, 2005) at levels comparable with adiponectin, one of the most abundant proteins in adipose tissue (Mracek *et al*, 2010a); ZAG, therefore, is a major adipokine. Recent evidence suggests that adipose tissue-derived ZAG is inversely linked to adiposity. *Zinc-α2-glycoprotein* gene and protein expression in adipose tissue is downregulated in obese *ob/ob* mice (Mracek *et al*, 2010b) and in obese human subjects (Selva *et al*, 2009). In contrast, ZAG mRNA and protein levels are substantially increased in adipose tissue of tumour-bearing mice with profound fat loss, suggesting a role for ZAG in adipose tissue catabolism (Bing *et al*, 2004).

Despite this knowledge, the clinical relevance of ZAG in human cancer cachexia is still not known. In this study, we investigated the potential role of adipokine ZAG in the pathogenesis of cancer cachexia in human beings. Zinc-*α*2-glycoprotein expression and secretion by adipose tissue (subcutaneous) was examined along with serum ZAG levels in cancer patients with cachexia as compared with weight-stable controls. We also analysed the relationship between adipokine ZAG (expression and release) and the extent of weight loss in cancer patients. Finally, the effect of recombinant ZAG protein on lipid mobilisation in human adipocytes was assessed.

## Materials and methods

### Patients and sample collection

Twenty-five cancer patients (upper gastrointestinal, *n*=12; pancreatic, *n*=13) were recruited at the Department of Clinical and Surgical Sciences, University of Edinburgh. Patients did not receive previous anticancer therapy and underwent potentially curative abdominal surgery. The weight and height of the subjects were measured, and body mass index (BMI) and total fat mass were calculated (Moses *et al*, 2001). Mid-arm muscle circumference (MAMC) was calculated from triceps skin-fold thickness (TSF) and total mid-arm circumference (MAC): MAMC=MAC−[*π* × TSF]. For each patient, pre-illness stable weight was self-reported and the percentage of weight loss was calculated. The patients were divided into cachectic (*n*=17) and weight-stable (*n*=8) groups. Cachexia was defined as unintentional weight loss ⩾5% during the previous 6 months; weight-stable patients were defined as patients without apparent weight change during the previous 6 months.

Patients had fasted overnight before undergoing surgery. A venous blood sample was taken before the surgical procedure and centrifuged to obtain serum, which was subsequently stored at −80°C for later analysis. An open biopsy of approximately 1–2 g of abdominal subcutaneous adipose tissue (SAT) was obtained at the start of the surgical procedure. Samples were rinsed in PBS and snap-frozen immediately in liquid nitrogen, and then stored at −80°C until later analysis.

For the study of ZAG protein release by adipose tissue, another cohort of 34 patients with gastrointestinal cancer (oesophagus, *n*=8; pancreas, *n*=12; stomach, *n*=4; gall bladder, *n*=2; and colon, *n*=5) and liver metastasis with no clear primary tumour (*n*=3) were recruited at the Karolinska University Hospital, Stockholm, Sweden. Among them, 15 patients experienced weight loss (⩾5% during the previous 6 months) and 19 were weight stable. The patients were investigated after an overnight fast. Height and weight was determined and body composition assessed by whole-body electrical bioimpedance (Quad Scan 4000; Bodystat, Isle of Man, UK). Abdominal SAT (∼500 mg) was taken by needle biopsy and immediately processed for measurements of *in vitro* protein secretions, as described previously (Ryden *et al*, 2008).

Tumour stage was assessed according to the AJCC/UICC system (Sobin, 2002). Fully informed, written consent was obtained in all cases and the study protocol was approved by the appropriate Regional Human Ethics Committees.

### Histology

Subcutaneous adipose tissue biopsies from cachexia and weight-stable cancer patients were fixed in 10% neutral formalin for 24 h, dehydrated in absolute ethanol, cleared in xylene and then embedded in paraffin. The paraffin was cut into 5-*μ*m sections that were stained with Harris haematoxylin, counterstained with eosin, and then evaluated and photographed under light microscopy. To detect the collagen-fibre content in SAT, sections were stained with Sirius Red (Bing *et al*, 2006).

### RNA extraction and real-time PCR

Total RNA was extracted from tissues or cells using Trizol (Invitrogen, Carlsbad, CA, USA). First-strand cDNA was reverse transcribed from 0.5 *μ*g of total RNA using an iScript first-strand synthesis kit (BioRad, Hercules, CA, USA) in a final volume of 10 *μ*l. Real-time PCR amplification was performed in a final volume of 12.5 *μ*l, containing cDNA (equivalent to 10 ng of RNA), primers, TaqMan probe FAM-TAMRA and a master mix made from qPCR core kit (Eurogentec, Seraing, Belgium) using a Stratagene Mx3005P instrument. The sequence of primers and probe for human ZAG, leptin and *β*-actin were as described previously (Bing *et al*, 2004; Bao *et al*, 2005). Polymerase chain reaction cycling conditions were: 95°C for 10 min, followed by 40 cycles (95°C for 15 s, 60°C for 1 min). Samples were normalised to *β*-actin and the results expressed as fold changes of *C*_t_ value relative to controls using the 2^−ΔΔ*C*_t_^ formula.

### Measurement of SAT ZAG protein

For quantification of ZAG protein levels, SAT samples were homogenised using glass/glass homogenisers in buffer consisting of 250 mM sucrose, 10 mM Tris-HCl (pH 7.4), 1 mM EDTA and protease inhibitor cocktail (dilution 1 : 500; Sigma, Poole, Dorset, UK). Homogenates were spun down at 12 000 **g** for 10 min at 4°C and upper fat layer was discarded. Infranatant and sediment were rehomogenised by pipetting up and down before being used for assay. The total protein content of the homogenates was determined by the BCA protein assay reagent (Sigma). Zinc-*α*2-glycoprotein protein content in tissue homogenates was measured with an enzyme-linked immunosorbent assay (ELISA) kit (BioVendor, Rosice, Czech Republic) according to the manufacturer's protocol. Zinc-*α*2-glycoprotein protein levels were normalised to total protein content.

### Measurement of SAT ZAG secretion

Subcutaneous adipose tissue explants were incubated as described previously (Ryden *et al*, 2008). In brief, adipose tissue (about 300 mg) was incubated in 3 ml of medium for 2 h at 37°C. The incubation medium was then collected from each sample and centrifuged at 1000 r.p.m. for 10 min to remove cell debris and the supernatant was stored at −80°C until analysis. The tissue was collected and subjected to lipid extraction. Zinc-*α*2-glycoprotein secretion by adipose tissue was determined as the protein concentration in the medium using an ELISA kit (BioVendor, Rosice, Czech Republic) and related to the lipid weight of the incubated tissue.

### Serum analyses

Serum ZAG levels were measured using an ELISA kit (BioVendor). Serum leptin levels were also evaluated by ELISA (R&D Systems, Abingdon, UK). The concentrations of serum albumin and lipids were determined by standard methods employed by the clinical chemistry laboratories.

### Culture of adipocytes

SGBS (Simpson–Golabi–Behmel syndrome) cells, kindly provided by Professor M Wabitsch, were maintained and cultured as described previously (Bao *et al*, 2005). In brief, cells were cultured at 37°C in a humidified atmosphere of 5% CO_2_/95% air. SGBS preadipocytes were maintained in DMEM/Ham's F12 (1 : 1) medium (Invitrogen, Paisley, UK) containing 10% (v v^−1^) foetal calf serum (FCS; Sigma) and antibiotics (penicillin/streptomycin; Lonza, Twekesbury, UK). Preadipocytes were seeded onto 12-well plates and grown until confluence. At confluence, cells were induced to differentiate (day 0) by incubation for 5 days in FCS free medium containing 0.25 *μ*M dexamethasone, 500 *μ*M 3-isobutyl-1-methyl-xanthine, 10 nM insulin, 200 pM triiodothyronine (T_3_), 1 *μ*M cortisol (all from Sigma) and 2 *μ*M rosiglitazone (GlaxoSmithKline, Uxbridge, UK). Cells were then maintained in feeding medium (containing 10 nM insulin, 1 *μ*M cortisol and 200 pM T_3_, all from Sigma) until full differentiation, verified by observing the accumulation of lipid droplets under the microscope.

For the study of the effect of ZAG on lipolysis, differentiated SGBS adipocytes (day 15) were treated with 5 or 20 *μ*g ml^−1^ recombinant ZAG (BioVendor) for 18 h. Isoproterenol (10 *μ*M) or IBMX (100 *μ*M) was similarly incubated with the cells as positive references. Cells without the addition of any agents were used as controls. At the end of incubation, the culture medium was collected and lipolysis was determined as glycerol release into the medium using a commercial kit (Sigma).

### Statistical analysis

Differences between the two groups were analysed by Student's unpaired *t*-test for normal distributed data. Mann–Whitney test was used for data with a non-normal distribution and data were presented as median with 75th percentile. Non-parametrical *χ*^2^ test was performed to compare difference of the tumour stage between the two groups. Associations between quantitative variables were assessed with Spearman's rank correlation test. Statistical analyses were performed using SPSS program, and statistical difference was considered as significant when two-tailed *P*<0.05.

## Results

### Characteristics of patients

The clinical characteristics of patients (the UK cohort and the Swedish cohort) are presented in Table 1. There were no significant differences in gender or age between cachectic and weight-stable cancer patients. Cachectic patients had a lower BMI (*P*=0.0015, UK cohort), fat mass (*P*=0.01–0.016, both cohorts) and MAMC (*P*=0.0082, measured only in the UK cohort), but greater weight loss (*P*<0.0001, both cohorts) compared with weight-stable patients. With respect to tumour stage, no significant difference was found between the two groups (both cohorts).

There was no significant difference in the serum concentration of ZAG between cachectic and weight-stable cancer patients (55.3±10.9 *vs* 58.7±7.7, *P*=0.45) (Table 1). Serum ZAG levels showed no correlation with BMI (*r*=0.092, *P*=0.66) or weight loss (*r*=−0.11, *P*=0.61). In contrast, serum leptin levels were lower (by 53%, *P*=0.035) in cachectic patients compared with weight-stable cancer patients (Table 1). Serum leptin also showed a positive correlation with BMI (*r*=0.61, *P*=0.0018) and an inverse correlation with weight loss (*r*=−0.53, *P*=0.0098).

### Adipose tissue remodelling in patients with cancer cachexia

**Figure 1:**
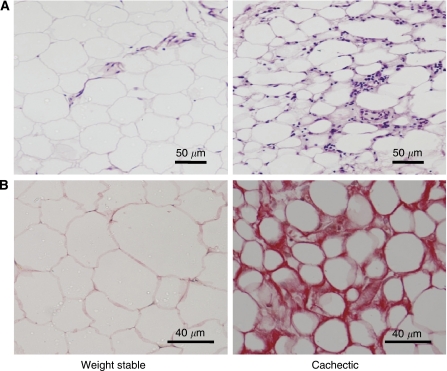
Morphological characteristics of adipose tissue in cachexia. Representative sections of subcutaneous adipose tissue from weight-stable and cachectic cancer patients stained with haematoxylin–eosin (**A**) or Sirius Red (**B**).

Examination under light microscopy showed substantial morphological alterations of SAT in cachectic cancer patients compared with weight-stable cancer controls. The changes were characterised by the tissue containing shrunken adipocytes and this was paralleled by an increase in interstitial space (Figure 1A). To further examine the nature of the extracellular matrix, sections of SAT were stained with Sirius Red. There was a strong Sirius Red staining in the stroma of adipose tissue from cachectic patients, which indicates a substantial increase in collagen-fibre content in the tissue (Figure 1B).

### ZAG expression in SAT is upregulated in cachectic cancer patients

**Figure 2:**
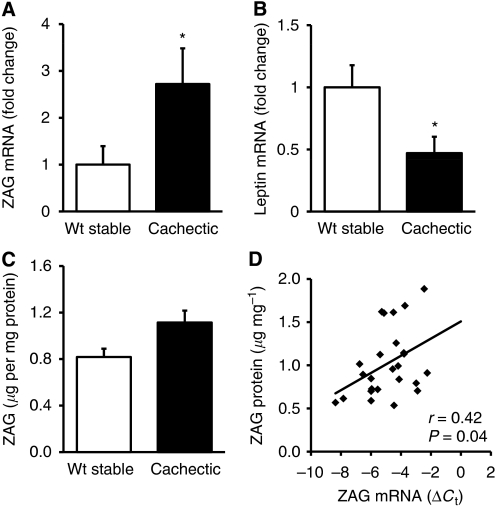
*Zinc-α2-glycoprotein* gene and protein expression in adipose tissue of cancer patients. mRNA levels of ZAG (**A**) and leptin (**B**) in subcutaneous adipose tissue of weight-stable (*n*=8) and cachectic (*n*=17) cancer patients by real-time PCR, expressed as means±s.e.m. Zinc-*α*2-glycoprotein protein levels in subcutaneous fat of weight-stable (*n*=8) and cachectic (*n*=17) cancer patients by ELISA, expressed as means±s.e.m. (**C**) Correlation between ZAG protein and mRNA levels (**D**) in adipose tissue; *n*=25. ^*^P<0.05 compared with weight-stable patients.

Subcutaneous adipose tissue ZAG mRNA levels were significantly higher in cachectic cancer patients than in weight-stable cancer patients (2.7-fold, *P*=0.028) (Figure 2A). In contrast, mRNA levels of leptin, known as an indicator of adiposity and here used as a reference gene for ZAG, were significantly reduced in cachectic patients (2.2-fold, *P*=0.018) (Figure 2B). Further analysis of ZAG protein expression in SAT showed an increase in cachectic patients compared with weight-stable cancer patients, but this was not statistically significant (1.4-fold, *P*=0.076) (Figure 2C). In addition, ZAG protein levels exhibited a positive correlation with ZAG mRNA levels in SAT (*r*=0.42, *P*=0.04) (Figure 2D).

### ZAG expression in SAT is associated with weight loss

**Figure 3:**
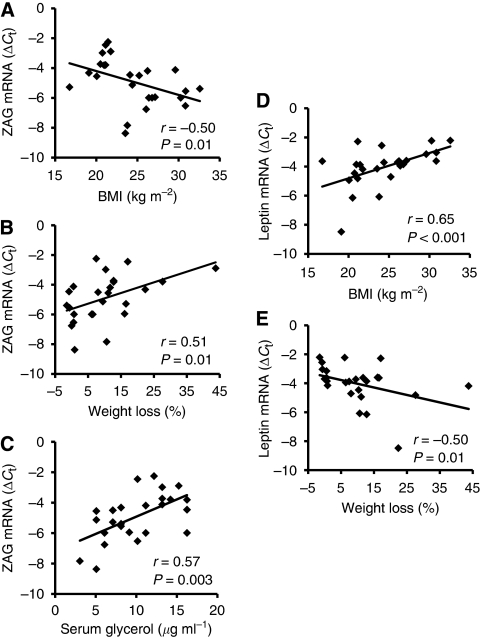
Correlation between ZAG mRNA levels in adipose tissue and BMI (**A**), weight loss (**B**) and serum glycerol (**C**); *n*=25. Correlation between leptin mRNA levels in adipose tissue and BMI (**D**) and weight loss (**E**); *n*=25. mRNA levels are presented as Δ*C*_t_ relative to *β*-actin.

To further investigate whether adipokine ZAG has a potential role in the pathogenesis of cancer cachexia in human beings, the relationship between SAT ZAG mRNA levels and several aspects of nutritional status were analysed. As shown in Figure 3A, ZAG mRNA levels correlated negatively with BMI (*r*=−0.50, *P*=0.01). In contrast, ZAG mRNA showed a positive correlation with weight loss (*r*=0.51, *P*=0.01) (Figure 3B). Furthermore, analysis of the potential link between adipokine ZAG and lipolysis revealed that ZAG mRNA levels correlated positively with serum glycerol levels (*r*=0.57, *P*=0.003) (Figure 3C). In addition, SAT ZAG protein levels also showed an inverse relationship with BMI (*r*=−0.41, *P*=0.045) and positive correlation with weight loss (*r*=0.41, *P*=0.04).

Contrary to the results of ZAG, leptin mRNA level showed a positive correlation with BMI (*r*=0.65, *P*<0.001) (Figure 3D), but were negatively correlated with weight loss (*r*=−0.50, *P*=0.01) (Figure 3E).

### ZAG protein secretion from SAT is elevated in cachectic cancer patients

**Figure 4:**
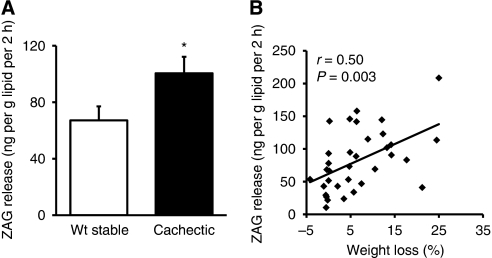
Zinc-*α*2-glycoprotein protein secretion by adipose tissue explants of cancer patients. (**A**) ZAG release was measured as the protein concentration in the medium of subcutaneous adipose tissue explants of weight-stable (*n*=19) and cachectic (*n*=15) cancer patients, expressed as means±s.e.m. (**B**) Correlation between ZAG secretion levels and weight loss; *n*=44. ^*^P<0.05 compared with weight-stable patients.

As there is enhanced ZAG expression in SAT in cancer cachexia, we investigated whether there is an increase in ZAG protein release from the tissue. Zinc-*α*2-glycoprotein was secreted by the SAT explants at levels of about 10–210 ng per g lipid per 2 h. In line with the results of ZAG expression, a 50% increase in ZAG protein secretion by adipose tissue was found in cachectic cancer patients when compared with weight-stable cancer patients (*P*=0.024) (Figure 4A). Furthermore, there was a positive correlation between ZAG protein secretion and weight loss in cancer patients (*r*=0.50, *P*=0.003) (Figure 4B).

### Treatment with ZAG stimulates glycerol release by human adipocytes

**Figure 5:**
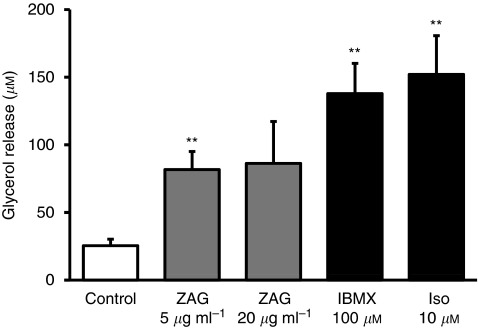
Effects of ZAG on glycerol release by human adipocytes. Differentiated SGBS adipocytes were incubated with recombinant ZAG (5 or 20 *μ*g ml^−1^), or isoproterenol (10 *μ*M) or IBMX (100 *μ*M) for 18 h. Control cells received no addition. Glycerol concentrations in the culture medium were determined. Data are means±s.e.m., *n*=4 per group. ^**^P<0.01 compared with controls.

In light of the upregulation of ZAG expression and secretion by adipose tissue in cancer cachexia, we investigated whether ZAG has a local effect through lipid mobilisation in human adipose tissue. Differentiated human adipocytes (SGBS) were treated with recombinant ZAG and the effect on lipolysis was examined. As shown in Figure 5, glycerol release by SGBS adipocytes was induced with a >3-fold increase at both the lower (5 *μ*g ml^−1^) and higher (20 *μ*g ml^−1^) doses of ZAG treatment, and the increase was statistically significant at the dose of 5 *μ*g ml^−1^ (*P*<0.01). As positive controls, treatment with isoproterenol and IBMX led to a six- and five-fold increase in glycerol release, respectively (both *P*<0.01) (Figure 5).

## Discussion

This study shows profound changes in SAT morphology in patients with cancer cachexia. Adipose tissue atrophy is characterised by a reduction in adipocyte cell size, and this is accompanied by a significant increase in tissue matrix fibrosis. Similar alterations in adipose tissue structure have been observed in our previous study in cancer-bearing mice (Bing *et al*, 2006). In agreement with these changes in adipose tissue morphology, a decrease in fat cell volume without change in total fat cell number has been recently reported in cachectic patients with gastrointestinal cancer (Agustsson *et al*, 2007; Ryden *et al*, 2008). These results provide evidence that adipose tissue remodelling with reduced lipid storage occurs in human cancer cachexia.

Until now, there has been no information available on ZAG production in cancer cachexia in human subjects. The results of this study show that the expression levels of ZAG transcripts and protein in SAT were upregulated in cachectic cancer patients compared with weight-stable cancer patients. The enhanced adipose tissue ZAG expression in cancer cachexia suggests that ZAG could be a local catabolic mediator within the tissue. Further analyses show that ZAG expression in SAT was linked to adiposity and weight loss in cancer patients. We show that ZAG mRNA levels exhibited an inverse relationship with BMI, but a positive correlation with weight loss. In parallel, ZAG protein content in adipose tissue correlated negatively with BMI, but positively with weight loss. Similarly, a negative association between adipose tissue ZAG expression and BMI has been reported in studies of obese human subjects (Selva *et al*, 2009; Mracek *et al*, 2010a). These observations indicate a contributory role for ZAG in the modulation of body adiposity (Bing *et al*, 2010). In this study, however, no significant differences in serum ZAG levels were found between cachectic and weight-stable cancer patients. Other data on circulating ZAG levels in relation to human obesity are inconsistent, being reported as either decreased (Ceperuelo-Mallafre *et al*, 2009; Selva *et al*, 2009) or increased (Yeung *et al*, 2009). Circulating levels of ZAG could be influenced by its production in other tissues such as the liver, a major site for ZAG production (Tada *et al*, 1991). Although ZAG mRNA levels were lowered in *ob/ob* mice (Mracek *et al*, 2010b) and obese patients (Selva *et al*, 2009), the extent of ZAG production by the liver in human cancer cachexia is not known and further studies are warranted. In addition, ZAG is known to be overexpressed by malignant tumours and this may over-ride the contribution of ZAG produced by adipose tissue to the circulating pool. Therefore, it is likely that adipose tissue-derived ZAG is more important locally through an autocrine/paracrine action.

In marked contrast to the results of ZAG, SAT leptin mRNA, which increases with fat mass accretion, is decreased in cachectic cancer patients. Furthermore, circulating leptin levels are also reduced in these patients. Leptin as a key adipokine is known to be an indicator of body adiposity (Kettaneh *et al*, 2007). The downregulation of leptin production in cachectic cancer patients is in agreement with a fall of adiposity in cancer cachexia.

Another key finding of this study is the demonstration that human adipose tissue secretes ZAG protein, and more importantly, the levels of ZAG secretion are higher in cachectic than in weight-stable cancer patients. Local secretion of ZAG from adipose tissue in cachexia has not been described previously. This study is the first to show upregulated production of the adipokine, suggesting that it plays a key autocrine/paracrine role in the cachexia-associated tissue atrophy. Importantly, further analyses reveal a positive relationship between ZAG protein release and weight loss in cancer patients. The elevation in ZAG production with increased severity of cachexia further supports a role for ZAG in catabolising body fat in human beings.

Although the molecular mechanisms of fat loss in cachexia remain to be established, increased lipolysis is considered to be a key factor. It has been shown that whole-body lipolysis, measured by the rate of appearance of glycerol, is higher in patients with cancer who are losing weight than in healthy subjects (Zuijdgeest-van Leeuwen *et al*, 2000). A recent study has shown that lipolytic activity (fasting plasma glycerol or fatty acids) is higher in cancer cachexia patients than in weight-stable controls (Agustsson *et al*, 2007). Furthermore, increased expression of hormone-sensitive lipase, a rate-limiting enzyme of the lipolytic pathway, has been reported in adipose tissue of patients with cancer cachexia (Thompson *et al*, 1993; Agustsson *et al*, 2007). *In vitro*, using mature adipocytes isolated from SAT, the lipolytic effects of catecholamines and natriuretic peptide were increased by >2-fold in cancer patients with cachexia compared with weight-stable cancer patients, although basal lipolysis was unaffected (Agustsson *et al*, 2007). In this study, we show a positive correlation between ZAG expression in adipose tissue and fasting serum glycerol levels, suggesting an involvement of ZAG in lipolytic activity.

The proposed role for ZAG in lipid mobilisation has primarily been based on studies in rodents and murine adipocytes (Hirai *et al*, 1998). Recent work has shown that ZAG-deficient mice are susceptible to weight gain when fed a high-fat diet, which appears to be the result of a decreased lipolytic response to adrenergic stimuli in white adipocytes, although basal lipolysis is not affected (Rolli *et al*, 2007). In ZAG-overexpressing transgenic mice, which exhibit decreased body weight and epididymal fat, mRNA levels of HSL in adipose tissue are elevated and the tissue is sensitised to the action of catecholamines (Gong *et al*, 2009). However, it is unclear whether this is the case in human beings with increased ZAG. We have shown that ZAG production (both expression and release) by SAT is increased in cachectic cancer patients, supporting a role for ZAG in human beings. Considering its action as a LMF in rodents, it is essential to examine further if ZAG acts as a catabolic mediator through its lipolytic effect in human adipose tissue. This study reveals that recombinant ZAG, at physiologically relevant concentrations, increases glycerol release from differentiated human adipocytes. Our finding suggests that ZAG could stimulate lipolysis in human adipose tissue, and therefore contribute to increased lipid mobilisation in human cancer cachexia. This result also provides the potential for novel therapeutic strategies against ZAG. The crystal structure of ZAG has been shown to have a high degree of similarity to the major histocompatibility complex class I molecules (Sanchez *et al*, 1999). As the hydrophobic groove formed by the *α*1–*α*2 domain of ZAG molecule may be important in lipid metabolism (McDermott *et al*, 2006; Hassan *et al*, 2008), further studies are warranted to assess whether blocking of this binding site and/or local ZAG production can be used for preventing the loss of adipose tissue in malignancy. On the other hand, ZAG might be employed for the treatment of visceral obesity as it stimulates lipolysis via *β*_3_-adrenoceptors (Russell *et al*, 2004) and *β*_3_-adrenoceptor sensitivity is enhanced in response to noradrenaline in visceral fat of obese subjects (Lönnqvist *et al*, 1995).

In conclusion, this study shows that in human subjects, ZAG expression and its protein secretion by SAT are enhanced in cancer cachexia irrespective of tumour stage. This upregulation of adipose tissue-derived ZAG is associated with the severity of cachexia. However, circulating ZAG levels do not differ between cachectic and weight-stable cancer patients. Finally, recombinant ZAG stimulates glycerol release from human adipocytes. Overall, our results suggest that adipokine ZAG plays a key role in the pathogenesis of cancer cachexia in human beings, at least in part, through its lipid mobilising action in adipose tissue.

## Figures and Tables

**Table 1:** Anthropometric, biochemical and hormonal characteristics of cancer patients

	**UK cohort**			**Swedish cohort**		
	**Weight stable (*n*=8)**	**Cachectic (*n*=17)**	***P*-value**	**Weight stable (*n*=19)**	**Cachectic (*n*=15)**	***P*-value**
Males/females (*n*)	7/1	8/9	0.088^*^	14/5	13/2	0.35^#^
Age (years)	67±6	64±10	0.69	66±7	64±11	0.49
BMI (kg m^−2^)	28.0±3.4	22.8±3.4	0.0015	25.7±3.5	23.3±4.2	0.072
Total fat mass (kg)	25.2±4.4	18.1±6.4	0.010		N/A	
Body fat (%)	30.8±3.7	27.1±4.8	0.060	27.3±3.7	21.7±8.4	0.0016
MAMC (cm)	26.5±2.9	23.2±2.4	0.0082		N/A	
Weight loss (%)	0.3 (0.7)	11.8 (16.8)	<0.0001^†^	1.2±2.7	13.3±6.3	<0.0001
						
*Tumour stage*						
1	3	3	0.26^#^	2	1	0.28^#^
2	0	6		0	0	
3	2	3		8	11	
4	2	3		8	3	
Albumin (g l^−1^)	36.9±3.9	32.7±5.4	0.064	37.9±2.6	34.0±3.7	0.0017
Triglycerides (mmol l^−1^)	1.28±0.74	1.26±1.04	0.96	1.20±0.35	1.08±0.42	0.37
Cholesterol (mmol l^−1^)	4.94±1.92	4.01±1.30	0.16	4.85±1.09	4.47±1.16	0.35
Glycerol (*μ*g ml^−1^)	0.44±0.25	0.64±0.37	0.19		N/A	
Total fat mass (kg)						
Serum ZAG (*μ*g ml^−1^)	58.7±7.7	55.3±10.9	0.45		N/A	
Serum leptin (ng ml^−1^)	13.7±6.0	6.6±7.3	0.035		N/A	

Abbreviations: BMI=body mass index; MAMC=mid-arm muscle circumference; N/A=not applicable; ZAG=Zinc-*α*2-glycoprotein.

Tumour stage was not available on one weight-stable patient from the Swedish cohort.

Values are means±s.d. or median (75th percentile). They were compared by either an unpaired Student's *t*-test, *χ*^2^ test (^#^), Mann–Whitney test (^†^) or Fisher's exact test (^*^).
